# Psoriasis comorbid with atherosclerosis meets in lipid metabolism

**DOI:** 10.3389/fphar.2023.1308965

**Published:** 2023-12-11

**Authors:** Liuping Chen, Huiqi Chen, Sien Guo, Zhijun Chen, Haifeng Yang, Yanjiao Liu, Xiaoling Chen, Xinming Chen, Tingting Du, Xinyao Long, Jiaxiong Zhao, Mingli Guo, Tianfeng Lao, DongHui Huang, Lei Wang, Jing Chen, Chunping Liu

**Affiliations:** ^1^ Department of Critical Care Medicine, The Dongguan Hospital of Guangzhou University of Chinese Medicine, Dongguan, China; ^2^ State Key Laboratory of Dampness Syndrome of Chinese Medicine, The Second Affiliated Hospital of Guangzhou University of Chinese Medicine, Guangzhou, China; ^3^ Second Clinical Medical College, Guangzhou University of Chinese Medicine, Guangzhou, China; ^4^ Affiliated Zhuhai Hospital, Southern Medical University, Zhuhai Hospital of Integrated Traditional Chinese and Western Medicine, Zhuhai, China; ^5^ Department of Cardiovascular Medicine, Guangdong Provincial Hospital of Traditional Chinese Medicine, Guangzhou, China; ^6^ School of Biotechnology and Health Sciences, Wuyi University, Jiangmen, China; ^7^ State Key Laboratory of Quality Research in Chinese Medicine, Institute of Chinese Medical Sciences, University of Macau, Macau, China; ^8^ Guangdong-Hong Kong-Macau Joint Lab on Chinese Medicine and Immune Disease Research, Guangzhou, China

**Keywords:** comorbidity, lipid metabolism, psoriasis, atherosclerosis, inflammation

## Abstract

Psoriasis (PSO) is a common skin disease affecting approximately 1%–3% of the population, and the incidence rate is increasing yearly. PSO is associated with a dramatically increased risk of cardiovascular disease, the most common of which is atherosclerosis (AS). In the past, inflammation was considered to be the triggering factor of the two comorbidities, but in recent years, studies have found that lipid metabolism disorders increase the probability of atherosclerosis in patients with psoriasis. In this review, we discuss epidemiological studies, clinical treatment methods, risk factors, and lipid metabolism of psoriasis and atherosclerosis comorbidities.

## 1 Background

Psoriasis is a common disease, with approximately 150,000 new cases of psoriasis reported each year, with recent studies showing an upward trend over the past 3 years. The prevalence of psoriasis in the population is between 2% and 3% (Valacchi et al., 2010). The data indicated that the occurrence of psoriasis varied according to age and geographic region, being more frequent in countries more distant from the equator (Parisi et al., 2013). The prevalence of psoriasis is affected by genetic, viral, endocrine and psychological factors (Harden et al., 2015). In recent years, psoriasis has been identified as a systemic disease associated with multiorgan abnormalities and complications. In patients with psoriasis, an increased risk of cardiovascular disease with atherosclerosis has been noted (Masson et al., 2020). Most research experts believe that psoriasis is an autoimmune disease, and the main mechanism is mediated by T cells. The early pathogenesis of skin lesions is chronic infiltration dominated by CD4^+^T lymphocytes, and the pathogenesis of late stages is slightly different from that of the early stage, mainly infiltrated by CD8^+^T lymphocytes, but the specific pathogenesis is still unclear (Cabrijan et al., 2009).

Recent studies have found that abnormal fat metabolism is an important factor in the pathogenesis of psoriasis. Patients with psoriasis will have significantly abnormal blood lipids and an increased risk of cardiovascular atherosclerosis (Uyanik et al., 2002). Common clinical dyslipidemia, including various lipid metabolism abnormalities, including high-density lipoprotein (HDL) reduction, is a major risk factor for cardiovascular diseases. In fact, Apolipoprotein A-1 (ApoA-1) constitutes the principal protein fraction of HDL, which plays a protective role in atherosclerosis by reversing cholesterol transport and so on. However, some recent studies have suggested different perspectives (Vuilleumier et al., 2013). They found that during chronic systemic inflammation, HDL could lose some of its atheroprotective functions, become dysfunctional or even proinflammatory. It can be seen that in the context of systemic inflammation, the mechanisms driving ApoA-1 and HDL towards pro- or anti-inflammatory molecules still needs to be studied. (Vuilleumier et al., 2013). Dyslipidemia is a risk factor for cardiovascular disease and an important cause of chronic inflammation and tissue damage, mediating the occurrence of atherosclerosis (Pirillo et al., 2021). The infiltration and retention of lipoprotein-containing Apolipoprotein B (ApoB) in the arterial wall is a key initiating event that initiates the inflammatory response and promotes the development of atherosclerosis. Arterial injury leads to endothelial dysfunction, promoting modification of lipoprotein-containing ApoB and infiltration of monocytes into the subendothelial space (Sniderman et al., 2019). Internalization of lipoprotein-containing ApoB by macrophages promotes foam cell formation, a hallmark of the fatty streak phase of atherosclerosis. Macrophage inflammation leads to oxidative stress and enhanced cytokine/chemokine secretion, resulting in more low-density lipoprotein (LDL)/residual oxidation, endothelial cell activation, monocyte recruitment, and foam cell formation. HDL, ApoA-1 and endogenous Apolipoprotein E (ApoE) prevent inflammation and oxidative stress and promote cholesterol efflux to reduce lesion formation (Libby et al., 2019).

## 2 Epidemiological study of atherosclerosis and psoriasis

Epidemiological studies have also confirmed the association between psoriasis and atherosclerosis. In 1973, a study of 300 hospitalized patients demonstrated for the first time that compared with other skin disease control groups, the risk of adverse cardiovascular disease (CVD) outcomes in patients with psoriasis was 2.2 times that of theirs (Caiazzo et al., 2018). The study first found that psoriasis was associated with the linkage of CVD risks. The first systematic review of 40,000 psoriasis patients in 1995 found that psoriasis was associated not only with cardiac insufficiency but also with diabetes and obesity, which are traditionally thought to cause AS (Henseler and Christophers, 1995). Subsequently, although some cohort studies concluded that psoriasis was not significantly associated with an increased risk of CVD (Miller et al., 2013) or there was only a slight association in severe psoriasis (Horreau et al., 2013), most of the subsequent systematic reviews supported that psoriasis is significantly correlated with AS-induced diseases such as myocardial ischemia and stroke (Frieder and Ryan, 2016; Sajja et al., 2018). Psoriasis and CVD risk have also been proven to present a “dose effect”; that is, CVD risk is positively correlated with the severity of psoriasis, and the study also found that psoriasis may be an independent risk factor for coronary atherosclerosis (Boehncke, 2018). However, some studies in recent years have drawn different conclusions. After correcting for traditional risk factors, there was no correlation between psoriasis and the risk of CVD adverse events. That is, the CVD risk in patients with mild psoriasis is more dependent on traditional risk factors. Only in patients with severe psoriasis was the adjusted risk association of psoriasis and CVD adverse events highlighted (Osto et al., 2012). Evidence has also found that psoriasis may be positively correlated with other traditional CVD risk factors, such as obesity, hyperlipidemia, and hypertension, but various conclusions are still inconsistent (Katsiki et al., 2014).

### 2.1 Risk factors for comorbidities

Smoking, excessive drinking, obesity and other unhealthy lifestyles are risk factors for cardiovascular complications in patients with psoriasis. A retrospective study found that the probability of cardiovascular disease in smoking patients was 1.78 (95% CI, 1.52–2.06), which was higher than that in nonsmoking patients (Armstrong et al., 2014). Excessive drinking is more likely to cause cardiac complications, such as atrial fibrillation, cardiomyopathy and sudden death. In addition, psoriasis is clearly associated with central obesity and increased abdominal visceral fat, which are important risk factors for cardiovascular disease (Owczarczyk-Saczonek and Nowicki, 2015). Studies have proven that obesity not only leads to vascular inflammation but also exacerbates the development of the disease and the upregulation of C-reactive protein (CRP), leptin, resistin, etc., increasing the risk of atherosclerosis (Henning, 2021). Changing the lifestyle of patients with psoriasis is beneficial to reduce the prevalence of cardiovascular complications (Tablazon et al., 2013). Age is also associated with the comorbidity of psoriatic atherosclerosis. Many studies have divided psoriasis into early-onset and late-onset psoriasis with an age cut off of 40 years old. Early-onset psoriasis has the highest risk of atherosclerotic complications, which may be related to the degree of physical involvement and systemic inflammation (Cozzani et al., 2018). In one study, patients with early-onset psoriasis had significantly elevated serum tumor necrosis factor-α (TNF-α) levels, higher levels of systemic inflammation, elevated serum endothelin levels, and carotid intima-media thickness, suggesting more severe endothelial dysfunction and a higher risk of developing atherosclerosis (Elkamshoushi et al., 2019).

In summary, patients with psoriasis have a higher risk of developing coronary atherosclerosis. Early detection, avoidance of possible risk factors, and correct treatment are helpful for the positive development of the disease, but more research is still needed to find a more suitable diagnosis and treatment method.

### 2.2 The relationship between lipid metabolism and psoriasis and atherosclerosis

**FIGURE 1:**
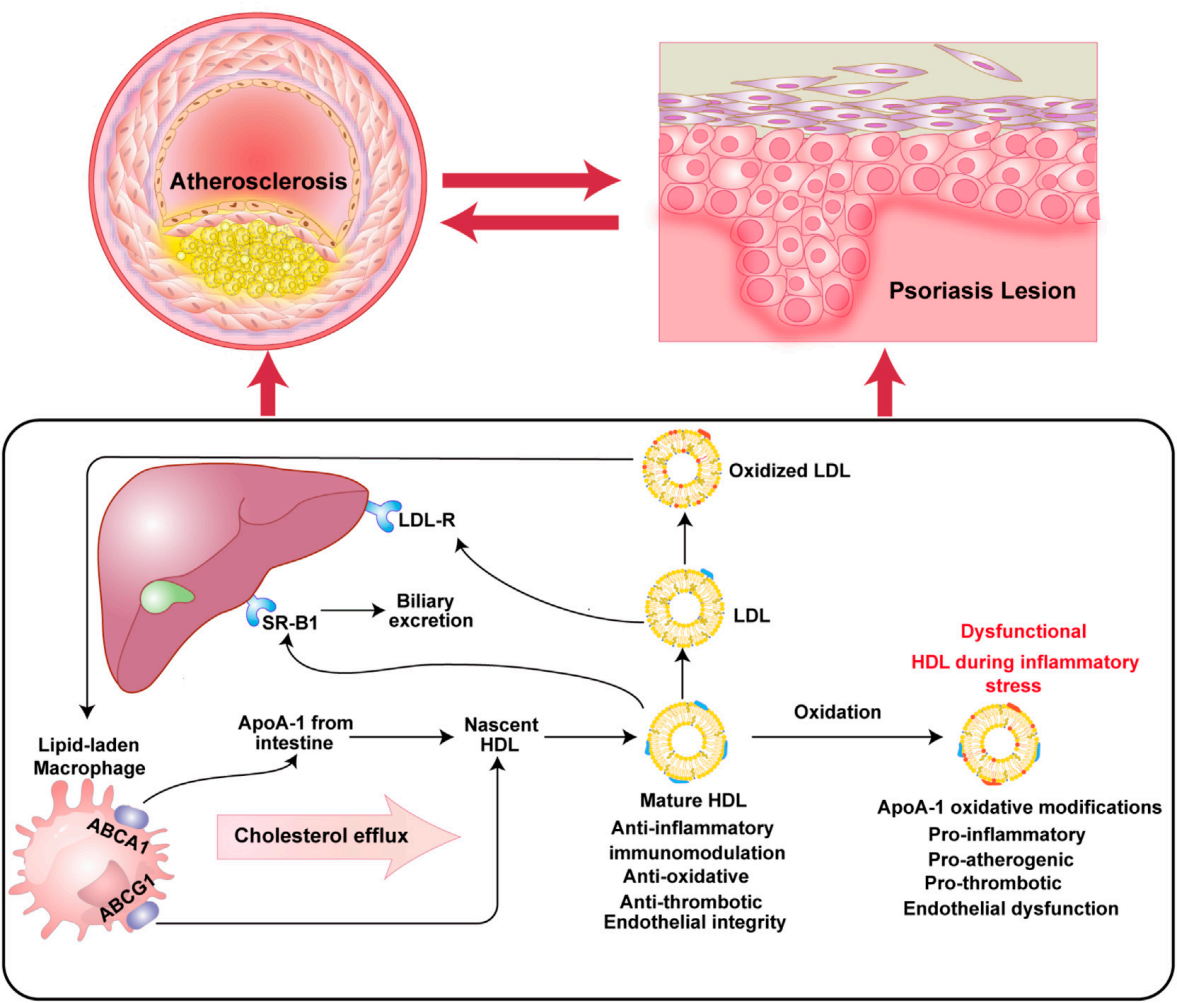
Abnormal lipid metabolism is the common pathogenesis of atherosclerosis and psoriasis.

Abnormal fat metabolism is considered to be an important factor in the pathogenesis of psoriasis. Patients with psoriasis will have significantly abnormal blood lipids and an increased risk of cardiovascular atherosclerosis (Nowowiejska et al., 2021). Apolipoprotein ApoA-1 is the main protein component of HDL, which not only regulates cholesterol transport to prevent cardiovascular disease but also participates in the regulation of inflammation and the immune response. Lipid metabolism depends on the regulation of plasma apolipoprotein ApoA-1. Dyslipidemia is a risk factor for cardiovascular disease and a cause of chronic inflammatory tissue damage, which together mediate the occurrence of atherosclerosis (van der Vorst, 2020) (Figure 1).

#### 2.2.1 Relationship between lipid metabolism and psoriasis

Metabolic syndrome characterized by lipid metabolism disorder is one of the important comorbidities in patients with psoriasis. This may be due to changes in serum cholesterol efflux capacity and macrophage cholesterol efflux mechanisms associated with atherosclerosis, mainly in blood lipid components. It is characterized by an increase or decrease in lipoproteins and changes in the ratio of other pure lipid components, such as triglycerides (Hao et al., 2021).

Metabolic syndrome is one of the important comorbidities of psoriasis. Approximately 20%–50% of psoriasis patients are affected by metabolic syndrome. It is a general term for metabolic disorders, and the diseases caused by them, including abdominal obesity, diabetes, hyperlipidemia, hypertension, and obesity-related nonalcoholic fatty liver disease, are closely related to lipid metabolism (Nowowiejska et al., 2021). A new single-center prospective study investigated the prevalence of metabolic syndrome in 60 children aged 3–10 years with psoriasis using the 2014 European Reference Standard for Metabolic Syndrome in Children. Compared with the incidence of metabolic syndrome in the general population, the risk of metabolic syndrome in children with psoriasis is significantly higher (30% vs. 5%) (Caroppo et al., 2021). This study complements the lack of epidemiological investigation of metabolic syndrome in young people and indirectly explains the high incidence of lipid metabolism disorders in psoriasis in all age groups.

Macrophages maintain the balance of normal lipid intake and excretion in lipid metabolism, and abnormal lipid metabolism can lead to abnormal deposition of lipids in macrophages (Yan and Horng, 2020). Flow cytometry and fluorescence analysis showed that bone marrow-derived macrophages (BMDMs) of diseased mice showed increased uptake of zinc oxide low-density lipoprotein and acetylated low-density lipoprotein, and the mechanism may be that the mRNA levels of modified LDL receptors were significantly increased, resulting in increased transcription (Baumer et al., 2018). Research demonstrated that targeted disruption of lipid transfer protein results in massive accumulation of both neutral lipids and phospholipids in macrophages within multiple tissues, following administration of a high-fat and -cholesterol diet (Kennedy et al., 2005). These gene changes can lead to reduced cholesterol export and increased storage. Stored cholesterol further leads to disordered lipid metabolism under the action of inflammatory factors in the inflammatory environment of psoriasis, such as interferon gamma and tumor necrosis factor alpha, in the mouse model by changing the pH of lysosomes and free cholesterol load. It accelerates the formation of cholesterol crystals in endothelial cells (Baumer et al., 2020).

The role of bioactive lipid mediators (LMS) in the development and regression of psoriasis is still not elucidated (Sorokin et al., 2018). Lysophosphatidic acid (LPA) is considered an inflammatory lipid whose elevated levels can contribute to psoriatic plaque inflammation or lead to complications (Zeng et al., 2017). In a study on the analysis of plasma glycerophospholipid metabolism, the results of an analysis of plasma glycerophospholipid metabolism by ultrahigh-performance liquid chromatography-tandem quadrupole mass spectrometry showed that LPA was hemolyzed. The levels of phosphatidylcholine and phosphatidic acidwere significantly increased, while the levels of phosphatidylcholine and phosphatidylinositol were significantly downregulated (Zeng et al., 2017). Using the same method, another study on phospholipid metabolism in monocytes found that free 4-hydroxynonenal-Hisadducts have a high level in psoriasis vulgaris patients and show increased endogenous cannabinoids in lipid enzyme metabolism. Cyclooxygenase-1 (COX-1) and cyclooxygenase-2 (COX-2) activity are enhanced in patients with psoriasis vulgaris (Wójcik et al., 2019). There is currently a lack of research on the expression of COX in psoriasis using TNF-α inhibitors. However, research has shown that the down-stream effector PGE2 of COX-2 can inhibit the self-renewal of intestinal stem cells during tumor necrosis factor inhibitor (TNF-i) treatment, thereby compromising tissue remodeling and regeneration (Li et al., 2018). These lipid metabolites can enhance oxidative stress and lead to inflammation. The lesioned skin of psoriatic patients contained more arachidonic acid metabolites, such as 8-, 12-, and 15-hydroxyeicosatetraenoic acid, and more linoleic acid-derived LMS.

#### 2.2.2 Relationship between lipid metabolism and atherosclerosis

Research on the relationship between lipid abnormalities and the pathogenesis of AS is still a hotspot. The previous concept believed that the increase in LDL level is the promotion factor of AS pathogenesis, while the increase in HDL level is the avoidance factor of AS. Therefore, LDL-lowering treatment is the first choice for AS treatment and is one of the core methods (Crismaru et al., 2020). Studies in recent years have further explored the necessity of lowering LDL levels; studies have been conducted on the remaining AS risk components after LDL reaches the treatment target, such as exploring serum triglyceride levels, medium-density lipoprotein cholesterol levels, and apolipoprotein B (Ling et al., 2017). Elevated risk association for AS. There are also new studies that have explored the deep association between LDL levels and AS, such as the correlation between “residual cholesterol” and AS. Residual cholesterol may be a more accurate measure of AS risk (Sandesara et al., 2019). The treatment of AS with elevated HDL has been proposed before, but the mortality rate of AS in people with high HDL levels is still high (Kingwell et al., 2014). Harmfully, improving the cholesterol reverse transport efficiency of HDL, that is, improving the cardiovascular protective function efficiency of HDL, has become a new research direction (Beazer et al., 2020).

The implementation of LDL-lowering therapy has achieved great benefits (Brandts and Ray, 2023), but whether it is safe and effective to apply it to older patients, use higher lipid-lowering intensity, and lower LDL target concentration has not been confirmed. A high-quality meta-analysis examining the benefits and harms of further lipid lowering at LDL levels of 1.8 mmol/L (70 mg/dL) showed that for every 1 mmol/L (38.7 mg/dL) reduction in LDL, the relative risk of major vascular events was 0.79 (95% CI, 0.71–0.87; *p ≤ 0.001*). Both statins alone and nonstatins combined had significant curative effects, and there were no obvious adverse events. Lipid is 1.2 mmol/L (Sabatine et al., 2018). A retrospective review of LDL therapy also confirmed that lowering LDL levels reduces the risk of AS-related stroke, regardless of initial LDL levels (Baigent et al., 2005). In the META analysis study of the sick population over 75 years old, the benefit of lowering LDL in the elderly population was affirmed. A 1 mmol/LDL level reduction can reduce the risk of AS by 26%, and this benefit is the same as that under the age of 75. Benefits were not significantly different across populations (Gencer et al., 2020).

In addition, a study found that regardless of low-density lipoprotein cholesterol levels (Raposeiras-Roubin et al., 2021), triglyceride (TG) levels ≥150 mg/dL were associated with subclinical noncoronary atherosclerosis, which was significantly associated with arterial inflammation. In terms of treatment research, in the case of already using statin drugs, a large randomized double-blind controlled multicenter study found that the use of ethyl eicosapentaenoic acid to further reduce the concentration of serum TGs in patients with various AS-induced CVD and stroke was associated with a significant reduction in risk, with a 20% reduction in cardiovascular mortality (Bhatt et al., 2019). This finding further confirms the feasibility of combination therapy with other therapeutic targets for treatment.

The ratio or concentration of cholesterol in lipoproteins is associated with AS (Burnett et al., 2020). The cholesterol component found in intermediate-density lipoprotein (IDL) and very-low-density lipoprotein (VLDL) particles has been termed “residual cholesterol” (Yang et al., 2023) or defined as that found in triglyceride-rich lipoprotein cholesterol content (Burnett et al., 2020). The study found that compared with patients with low levels of residual cholesterol, patients with residual cholesterol ≥30 mg/dL (0.78 mmol/L) had a higher risk of AS, which was independent of low-density lipoprotein cholesterol (LDL-C) levels (Masson et al., 2017). In other experiments, it was also found that the level of residual cholesterol has nothing to do with the level of apolipoprotein B, and the correlation between LDL-C and CVD decreased after controlling for residual cholesterol (Johannesen et al., 2021). Therefore, it is speculated that residual cholesterol may be one of the underlying reasons why LDL-C is associated with the risk of atherosclerotic cardiovascular disease, and it is beneficial for us to evaluate CVD risk more accurately (Hoogeveen and Ballantyne, 2021).

For the method of raising HDL to treat AS, the study used the cholesteryl ester transfer protein inhibitor evacetrapib. The HDL level of the test group was significantly increased, the LDL level was significantly decreased, and the cell cholesterol efflux capacity was increased, but the end-point CVD risk was still not statistically significant compared with the control group difference (Lincoff et al., 2017). The explanation for this may be that HDL is produced ineffectively, although various hypotheses are not supported. The latest scavenger receptor class B type 1mouse model study may explain this result; that is, higher levels of HDL may cause free cholesterol to flow from HDL to LDL and macrophages with poor cholesterol efflux ability. In the case of changes, the increase in this speed can increase the free cholesterol content of multiple tissue cells in the human body (Liu et al., 2021). In the experiment, the model mice lost HDL. The ability of cholesterol to be excreted into bile (Hoekstra and Van Eck, 2015) shows that the increase in HDL levels does not necessarily play a protective role in AS, and the function of HDL also needs to be re-examined.

In addition, genetic studies have found that AS disease populations are more susceptible to AS related to lipid metabolism genes than normal populations. Loss-of-function variants in the angiopoietin-like 3 gene are associated with reduced levels of blood triglycerides, LDL-C, and HDL cholesterol, and this case‒control study found that angiopoietin-like 3 has less loss of gene function variation (adjusted odds ratio is 0.59; 95% confidence interval is 0.41–0.85; *p = 0.004*), which may lead to the inability to maintain a low level of blood lipids in this population, thereby aggravating AS (Dewey et al., 2017).

### 2.3 The clinical method of treating psoriasis and AS comorbidity

At present, the blood cholesterol management guidelines of the American Heart Association have clearly pointed out that chronic inflammatory diseases such as psoriasis are factors that enhance the risk of cardiovascular diseases such as atherosclerosis (Grundy et al., 2019). Studies have shown (Späh, 2008) that inflammation plays an important role in the link between psoriasis and coronary artery disease, so methotrexate (MTX), cyclosporin and TNF-i anti-inflammatory drugs play a key role in the treatment of comorbidities in psoriasis and AS (Shapiro et al., 2007) (Table 1). The guidelines of different countries are similar in the treatment of psoriasis complicated with coronary heart disease, but there are differences in the first choice of drugs (Shah et al., 2018). For example, the first-line system of France (Amatore et al., 2019) tends to prefer TNF as the preferred second-line systemic therapy over MTX. According to the United States (Elmets et al., 2019) combined care guidelines, patients with psoriasis treated with TNF is have a lower risk of major adverse cardiovascular events than those treated with methotrexate.

**TABLE 1:** Applications of drugs in comorbidities in psoriasis and AS.

State	Drug	Mechanism	Clinical application	Security	Ref
Marketed drugs	Methotrexate	Inhibiting purine, the adenosine pathway, chemotaxis and adhesion of inflammatory cells, proinflammatory cytokines and polyamine and lymphotoxin formation	Severe psoriasis and rheumatoid arthritis	Low-dose methotrexate reduces morbidity while long-term use can be toxic	Prodanovich et al. (2005), Verhoeven et al. (2021)
	Adalimumab	Reduce E-selectin VCAM-1, and IL-22	Psoriasis and cardiovascular disease comorbidities such as coronary heart disease, stroke, peripheral vascular disease	Not mention	Gkalpakiotis et al. (2017), Zdanowska et al. (2020)
	Etanercept	Reduce biomarkers of cardiovascular risk such as soluble VCAM-1, soluble ICAM-1, soluble E-selectin, MMP-9, MPO, and tPAI-1, decreased CRP levels	Psoriasis and cardiovascular disease	Not mention	Sigurdardottir et al. (2014), Campanati et al. (2015)
	Ustekinumab	Improve coronary and myocardial function; reduces serum protein levels associated with cardiovascular risk in psoriasis vulgaris, such as N-terminal prohormone brain natriuretic peptide	Psoriasis	Unknown	Reich et al. (2011), Koschitzky et al. (2022)
	Fumaric Acid Esters	Decrease serum CRP level and increase adiponectin level; decreased TC level and Apo-B level	Severe psoriasis	Unknown	Boehncke et al. (2011), Holzer et al. (2021)
	Cyclosporine	Inhibits T-cell-mediated responses	Psoriasis	Increased risk of hypertension, hyperlipidemia and nephrotoxicity	Griffiths and Voorhees (1990), Ryan and Kirby (2015)
Drugs under development	Briakinumab	Blocks the biological activity of cytokines IL-12 and IL-23	Psoriasis	Needs to be further evaluated	Traczewski and Rudnicka (2012)

Abbreviations: IL-22, Interleukin-22; VCAM-1, Vascular cell adhesion protein 1; ICAM-1, Intercellular adhesion molecule-1; MMP-9, Matrix metalloproteinase-9; MPO, myeloperoxidase; tPAI-1, total plasminogen activator inhibitor-1; CRP, C-reaction protein; IL-12, Interleukin-12; IL-23, Interleukin-23;TC, total cholesterol; Apo-B, Apolipoprotein B.

Regarding how to choose the first-line drug for the treatment of psoriasis and AS comorbidity, it has been pointed out (Yang et al., 2016) that compared with local treatment/phototherapy, methotrexate treatment with a TNF-α inhibitor was associated with a significantly lower risk of cardiovascular events compared with treatment with MTX. The risk of myocardial infarction was also reduced in psoriasis patients treated with TNF-α inhibitors compared with psoriasis patients treated with topical therapy and MTX (Shaaban and Al-Mutairi, 2018). In addition, long-term use of MTX leads to a risk of end-organ toxicity, whereas TNF-α inhibitors have a small risk of end-organ damage (Kaushik and LebwohlPsoriasis, 2019). Therefore, some experts believe that TNF-α inhibitors are the systemic drugs of choice for the treatment of psoriasis patients with cardiovascular risk factors. Wu and Poon reported (Wu and Poon, 2013) that the risk of myocardial infarction was substantially reduced in psoriasis patients treated with TNF-α inhibitors compared with untreated patients. At the same time, the abnormal effects of TNF-a antagonists on vascular function are also controversial (Dulai et al., 2012). A study found that the endothelial function and arterial elasticity of patients with moderate to severe psoriasis were improved after 6 months of adalimumab treatment (Pina et al., 2016). However, as there are few reports on how TNF-i affects COX, the specific mechanism of how adalimumab interferes via cyclooxygenase in preventing atherosclerosis remains to be explored. After 12 weeks of etanercept treatment in young patients with mild psoriasis, there was no significant change in endothelial function or vascular stiffness (Hayek et al., 2015). The reason for the difference. This suggests that more long-term data are needed on the therapeutic effect of TNF-α inhibitors.

With continuous in-depth research on psoriasis and cardiovascular diseases, new therapeutic targets have received more attention. Overall, epidemiological data suggest a positive or neutral impact on cardiovascular health for TNF, IL-17A and IL-12/23p40, but current evidence remains conflicting for anti-IL-23/p19 and JAK inhibitors (Weber et al., 2021). More research is needed to better assess the effect of biologic therapies on cardiovascular risk and to select more appropriate drugs for the treatment of psoriasis and AS comorbidities.

## 3 Summary

A large number of studies have explained the reasons for the comorbidity of psoriasis and coronary atherosclerosis from different angles, but there is still no clear mechanism. Psoriasis and AS have similar immune-mediated inflammatory responses, and it was previously believed that immune imbalance may be the common mechanism of both. However, increasing evidence shows that lipid metabolism disorders play an important role in the comorbidity of psoriasis and atherosclerosis. The association between psoriasis and AS may be the result of multifactorial interactions and is not limited to immune-inflammatory responses. In summary, literature reports and research results show that patients with psoriasis have an increased risk of developing central vascular diseases, lipid metabolism disorders may be the common pathogenesis of the two, and further research is needed.

## Abbreviations

AS, Atherosclerosis; HDL, High-density lipoprotein; ApoA-1, Apolipoprotein A-1; LDL, Low-density lipoprotein; ApoB, Apolipoprotein B; ApoE, Apolipoprotein E; CVD, Cardiovascular disease; CRP, C-reactive protein; TNF-α, Tumor necrosis factor-α; LMS, Lipid mediators; LPA, Lysophosphatidic acid; Cyclooxygenase, COX; COX-1, Cyclooxygenase-1; COX-2, Cyclooxygenase-2; TG, Triglyceride; IDL, Intermediate-density lipoprotein; VLDL, Very-low-density lipoprotein; LDL-C, Low-density lipoprotein cholesterol; MTX, Methotrexate; CSA, Cyclosporin.
